# Travel across time zones and the implications for human performance post pandemic: Insights from elite sport

**DOI:** 10.3389/fpubh.2022.998484

**Published:** 2022-12-02

**Authors:** Stephen C. Jasper, Mark A. A. M. Leenders, Tim O'Shannassy

**Affiliations:** Graduate School of Business and Law, RMIT University, Melbourne, VIC, Australia

**Keywords:** RMIT classification, management, time zones, jet lag, public health, performance

## Abstract

Notwithstanding technological innovation, the COVID-19 pandemic, and new communication tools, the need for travel is growing again and, in some travel segments, it is stronger than ever. Interestingly, the public health implications of traveling across time zones are still poorly understood and this is especially true for organizations that send their workers across the globe. Using data from 173 Olympic teams over 15 Olympic Games, we show that crossing multiple time zones has negative implications for human (sports) performance. More importantly, the results indicate that performance impairment is especially visible after flying east, with peak performance particularly impaired, leading to a “gold demotion effect” of gold medals to silver medals as a result. Given that Olympic sporting teams typically have dedicated medical staff and active mitigation strategies, these findings have important public health implications. For example, organizations are demanding their workers to be on “top of their game” while traveling, without providing them with the support and tools to do so. The implications for public health management and human resource management are discussed.

## Introduction

Globalization is an important driver for organizational growth and performance and to tap into opportunities for growth, organizations increasingly use global teams for innovation and market development. Notwithstanding the huge potential of employing global teams, there are organizational health risk factors that are poorly understood resulting in suboptimal outcomes from employees working across the globe and for public health in general (1).

Global organizational structures are increasingly facilitated through new technologies such as collaboration tools and video conferencing. This trend has seen tremendous growth as COVID-19 dramatically interrupted business travel and new ways of working using Zoom and other collaboration tools became part of the new normal (2). Although technology is facilitating new ways of communication and collaboration, team members continue to keep a significant travel schedule that is poorly understood at the individual level, let alone at the organizational level in terms of stress, health and human impact (3).

The purpose of this article is to investigate an area where more is known about jet lag and impact and use this setting to further our understanding at the organizational and community level. In the area of global elite sport, much more is known about athletes, their travel schedules, health and their performance (4). This provides an interesting opportunity for interdisciplinary research to answer a question in global movement and management research that “has overlooked an important category of international operator: the international business traveler” (3).

This study is not about sports *per se*, but it uses a non-traditional assignment arena of athletes to inform studies on business travel and its impact on global team performance (5). Crossing multiple time zones can result in jet lag and sleep deprivation (6), which are likely to have adverse effects on performance (6, 7). Athletes—and there is anecdotal evidence a similar approach exists for employees—will try to mitigate the negative impact of travel by flying in early to allow adjustment, or by using some form of jet lag treatment plan (8, 9). Strategies to ameliorate the effects of jet lag include light seeking or avoidance, melatonin, and other pharmacological agents (8, 10). Compared to the support that athletes get, workers who travel get little or no support, even for travelers who traveled internationally as many as 26 times a year (11).

Little is still known about the extent of the impact and duration of jet lag, including social jet lag, on performance, although the rule of thumb is that recovering from jet lag takes “a number of days equal to about two-thirds of the number of time zones that have been crossed” (9) and the impact and duration of these effects vary across individuals and occasions (7, 12). The potential performance impairment for global teams that develop their market or develop new innovations is poorly understood. In addition, there may be an important need to support traveling workers better when they fly longer distances (1).

There is growing evidence that the symptoms of jet lag can worsen before resolving and that the direction of flight also has an impact (6). The circadian rhythm is a complex mechanism mediated by light, and jet lag is a disruption of this circadian rhythm (6). The symptoms of jet lag include insomnia and daytime sleepiness, but can also include “dysphoric mood, diminished physical performance, cognitive impairment, and gastrointestinal disturbances” (6), and even urticaria (13).

In this study, the aim is to use data on the travel and performance of Olympic national teams to understand the performance of international business travelers and the implications on international human resourcing and global work practices. Because the effects of jet lag can have a substantial impact on physical and cognitive ability (6), it is important to investigate these effects and their impact more closely. We present multi-year evidence of a measurable and significant adverse effect of crossing multiple time zones on Olympic team performance, with an emphasis on medal achievement composition. The results provide evidence of a “demotion effect” where athletes who were likely to win a gold medal win a silver medal instead. This “demotion effect” is studied while controlling for a range of other factors. The findings have implications for both athletes and business travelers and for organizations, including Australian businesses where business travel is prolific given the geographical location.

The structure is as follows; first, the factors influencing Olympic team performance are discussed, and the potential role and impact of jet lag on performance is explored. Second, three hypotheses are proposed on the relationship between the number of time zones crossed and the direction of flight on performance. Thirdly, the data set covering 15 Olympic Games and the methodology used to analyze this data is outlined; the results are presented and discussed; conclusions are drawn, and the implications and limitations are analyzed. Finally, the significance of the results for theory, management, organizations, and travel are discussed.

### Factors influencing Olympic performance

The Olympic games provide the context for our study on the impact of travel on human performance (4). To ensure the best performance outcomes, athletes need to be at their peak in terms of ability, training, support, and preparation. Several studies have identified determinants of Olympic success and a range of factors have previously been used to predict success in the Olympic Games.

The population size of the country is an obvious factor for Olympic medal success: nations with larger populations have an increased statistical probability of producing Olympic medal-winning athletes than nations with a smaller population to draw from. This positive relationship between population and medal count has been confirmed by Putt (14), who analyzed the correlation between population and weighted medal count (where gold, silver and bronze medals were weighted in a 3:2:1 ratio). Accordingly, almost every model used to predict Olympic medal count uses population as a basic factor to predict Olympic medal performance. A similarly intuitive measure for predicting Olympic medal success is the nation's gross domestic product (GDP) and its economic factors, as wealthier nations have more resources to direct to Olympic performance than less wealthy ones, and almost all predictive models take into account a nation's GDP *per capita*. Thus, a nation's population multiplied by its GDP *per capita* is the usual starting point for calculating Olympic medal success. Nations that have a strong sporting culture will show Olympic results above what would be expected for their population and GDP *per capita* (e.g., Bulgaria, North Korea). Often politics and national sport organizations are involved to win national esteem and to invest and support athletes' performance (15). Finally, there are some Olympics specific factors such as the boost from hosting the Olympic Games and the strong positive effect on Olympic medal performance for the host nation is borne out of having a home advantage and incentives to invest (16). These necessary control factors will be included in our analyses.

#### Time zone disparity and jet lag

Jet lag typically occurs when a person crosses three or more time zones (9), has a negative effect on performance (17), which extends to performance in sports (18). The effect is typically worse flying east than flying west [(19, 20), pp. 136, 138], and the degree of jet lag can be assessed with standardized scales to quantify this (7, 12, 21).

The effect of jet lag has been documented and studied considerably (18), and the negative impact of jet lag has been reported by athletes themselves; in a series of 15 interviews with Olympic athletes, jet lag was noted as a factor that negatively impacted performance (22). For an overview of Jet Lag studies see Table 1. As a result of this effect from jet lag, comprehensive and detailed jet lag management plans specifically for athletes have been devised, depending on the number of time zones crossed and the direction of flight (8), as the degree of jet lag is proportional to the number of time zones crossed (6).

**Table 1:** Studies in jet lag.

**Study**	**Physical effect**	**Cognitive effect**	**Biological markers**	**Mood effect**	**Sleep effect**	**Sample size**	**Population studied**
Arendt et al. (40)	No	✓	✓	✓	✓	17	Healthy volunteers
Arendt and Aldhous (41)	No	No	✓	No	No	52	Long-distance travelers
Becker et al. (7)	No	✓	✓	✓	✓	89	Long-distance travelers
Belcaro et al. (42)^a^ (study 1)	No	✓	✓	✓	✓	68	Long-distance travelers
Belcaro et al. (42)^a^ (study 2)	No	✓	✓	✓	✓	65	Long-distance travelers
Cho et al. (43)	No	✓	✓	No	No	62	Airline cabin crew
Claustrat et al. (44)	No	✓	No	✓	✓	30	Business travelers
Eastman et al. (45)	No	No	✓	No	✓	26	Healthy volunteers
Edwards et al. (46)	✓	✓	✓	No	✓	31	Business travelers
Fowler et al. (47)	✓	No	✓	✓	✓	13	Healthy male volunteers
Fowler et al. (48)	No	No	✓	No	✓	18	Athletes
Hill et al. (49)^b^ (study 1)	✓	No	No	✓	✓	7	Athletes
Hill et al. (49)^b^ (study 2)	✓	No	No	✓	No	10	Healthy volunteers
Hill et al. (49)^b^ (study 3)	✓	No	No	No	No	9	Healthy volunteers
Jurvelin et al. (50)	No	No	No	✓	✓	55	Healthy volunteers
Katz et al. (51)	No	No	No	✓	No	152	Foreign tourists
Lahti et al. (52)	No	No	No	No	✓	15	Airline cabin crew
Lemmer et al. (24)	✓	No	✓	No	✓	19	Athletes
Monk et al. (19)	No	No	✓	No	✓	20	Healthy elderly volunteers
Nickelsen et al. (53)	No	No	✓	No	No	36	Long-distance travelers
Paul et al. (54)^b^ (study 1)	✓	✓	✓	No	No	14	Healthy volunteers
Paul et al. (54)^b^ (study 2)	✓	✓	✓	No	No	13	Healthy volunteers
Paul et al. (54)^b^ (study 3)	✓	✓	✓	No	No	10	Healthy volunteers
Petrie et al. (55)	No	No	✓	✓	✓	20	Healthy volunteers
Petrie et al. (56)	No	No	✓	✓	✓	52	Airline cabin crew
Sasaki (25)	No	No	No	No	✓	4	Healthy volunteers
Spitzer et al. (21)	No	No	✓	✓	✓	257	Physicians
Suhner et al. (57)	No	No	No	✓	✓	320	Healthy volunteers
Suhner et al. (58)	No	No	✓	✓	✓	137	Healthy volunteers
Waterhouse et al. (59)	✓	No	No	✓	✓	39	Athletes and sport administrators
Waterhouse et al. (18)	No	No	✓	No	✓	85	Athletes

a Belcaro et al. (42) and Zerbini et al. (60) include two studies.

b Hill et al. (49) and Paul et al. (54) each include three studies.

The effects of crossing multiple time zones and the resultant jet lag have been assessed in a variety of sports, such as basketball (23). However, while the athletic community has been studied heavily, research into the effect of jet lag on international business travelers has been overlooked, with a lack of organizational support for business travelers despite them being a valuable resource to their company (3, 4). International business travelers are often “conducting business in a cloud of caffeinated jet lag” (17), despite the duty of care owed by companies to their employees (11).

#### Olympic medal team performance

While travel time and mere distance may affect performance, this article makes the hypothesis that time zone disparity is an overlooked factor on athletic performance. Olympic medal tallies, in the Summer Olympics, are expected to be impacted in specific ways. As time zone disparity increases athletic performance is likely to be impacted (6). So there are a number of elements to the individual athlete's presentation for the personal examination presented by competition that are impacted adversely by time zone disparity, making high performance more difficult (1). Thus, it would seem highly likely that as the number of time zones crossed increases, the medal count decreases (i.e., human performance decreases).

***H1: There is a negative relationship between time zone disparity (the number*** ***of time zones crossed) and human sports performance*.**

The direction of flight matters with respect to crossing time zones and performance. Typically, flying east results in more severe jet lag than flying an equivalent distance west [(20), pp. 136, 138]. This finding was borne out in a study of 20 healthy elderly subjects aged 67–87 years who had their circadian rhythm advanced or delayed, as alertness was disrupted more after the phase advance than after the phase delay (19). However, it has been noted that some people may respond better to flying eastward (18).

In a study that compared jet lag experienced by athletes flying west vs. flying east, the athletes who flew east took a longer time to adjust to the new time zone (24). Re-entrainment into the destination's time zone is more difficult if the circadian rhythm is contracted, which happens as a result of flying east, and is generally slower after flying east than it is after flying west (6, 25). Additionally, the direction of flight correlates with the type of jet lag experienced, with flying west associated with evening sleepiness and extremely early awakenings and flying east with difficulties falling asleep and morning or noontime sleepiness [(20), pp. 136, 138]. Even in the American National Football League with its limited time zone disparity of 3 h from the east to the west coasts of the United States, it was found west coast teams had an advantage in evening games (26). Similarly, in the American sports context eastward flight has been shown to negatively impact performance in baseball more than the equivalent westward flight (27).

Thus, it would seem reasonable to infer that performance will be more adversely affected, and therefore have a lower medal count in the Olympics setting, than those who fly west or remain in the same time zone, despite the preparation that teams will have made (1).

***H2: There is a more negative relationship between time zone disparity and*** ***human sports performance if the travel is in easterly direction*.**

#### Peak performance impact

Medical jet lag research has often focused on physical performance using objective biological markers such as vitamins or minerals (28); trace elements or biological variables such as nitric oxide (29). These indicators have been shown to have limited validity in terms of actual performance, with self-reported measures of physical performance being more useful than objective biological markers (30).

Human performance is not only dependent on physical attributes such as fine motor skills, gross motor performance but senses including eyesight, hearing, reflex response and cognitive function as well can all be important on the outcome depending on the sport (31). Thus, effective predictors of athletic performance include measures of physical and cognitive performance in preference to biological markers.

Peak performance has been shown to be closely aligned to the “flow” state described by Csikszentmihalyi (32). Given the disruption to cognitive function that can be caused by crossing multiple time zones and the resultant jet lag (6), it would seem reasonable to infer that the ability to attain peak performance could be impaired significantly by jet lag.

In order to obtain a gold medal, peak performance is paramount. The difference between gold and silver medals can be extraordinarily small; if a competitor traveled across more time zones than their competitors there may be a relatively small impairment of athletic performance, but it will result in a different medal. This small impairment may be very significant in terms of Olympic medal count: “at Athens, the combined margin of five British gold medals being silver medals was only 0.545 s—that shows how close it can be” (33). Thus, even the slightest effect on peak performance is likely to affect the gold medal count, demoting a competitor to silver or even bronze.

The small but significant deterioration in athletic peak performance may result in the competitor or team missing out on receiving a gold medal but receiving a silver medal or less instead. This “demotion effect” would be apparent if the mix of medals is downgraded. If only the highest levels of peak performance are affected, it is possible the lower medals (silver and bronze) may avoid this “demotion effect” and even get boosted instead.

Several studies support the notion that peak performance may suffer most. For example, it is widely known that a person's chronotype affects the time during the day where performance peaks [(20), pp. 152–162]. For athletes, the variation in performance according to the time of day does have some endogenous input, most likely genetic (31).

***H3: There is a negative relationship between crossing time zones in easterly*** ***direction and human performance. This relationship is more negative for peak*** ***performance*.**

For hypothesis 3 to hold the team's gold medal tally needs to show a “demotion effect” in gold medals (reflective of peak performance) and it may or may not show an increase in the tally of the lower medals (silver, bronze), depending on whether the performance impairment is more homogeneous across the performance curve. If there is a boost in silver medal count and not in bronze, it shows that silver medal count is less impaired then gold and also that silver “demotion” is not affecting the bronze medal tally significantly.

If there is support for (some of) the hypotheses in the highly performance managed arena of the Olympic Games, there are significant implications for the “amateurs” and unsupported team members in most organizations that have adopted global teams to compete better. The method, data and results will be presented next.

## Methodology

The data set covers 15 Olympic Games, starting with the Games of the XVII Olympiad held in Rome, 1960 until the Games of the XXXI Olympiad held in Rio de Janeiro, 2016. The Olympic Games held in 1960 was chosen as the start of the data set as jet airliners did not replace rail and ocean liners as the primary mode of long-distance travel until 1958 (16). Data were taken directly from the International Olympic Committee (IOC) website. For the purpose of comparability and control, only data from the Summer Olympics (as opposed to the Winter Olympics) were used. For data on populations and GDP, Google Public Data was used. Medals that were awarded and later revoked are not included in the data set. In terms of the sampled nations, only the 15 leading nations with stable borders were included. This approach makes sure that the countries are relatively similar but can still face significant different time zone challenges across different games.

### The dependent variables

The success on Olympic Games is measured in medals. Interestingly, there is additional detail in terms of the type of medal. *Gold medals (total)*: The number of gold medals won by a country. *Silver medals (total)*: The number of silver medals won by a country. *Bronze medals (total)*: The number of bronze medals won by a country. *Total medals*: The total number of medals won by a country.

### The independent variables

#### Absolute time zone difference

For absolute time zone difference (ATZD), the difference of the time zone of the capital city of the competing nation and of the host city was used, according to the most direct route between the two cities. The maximum amount of time zone disparity that can occur is 12 h, and daylight saving is not taken into account due to the circadian rhythm being linked to “sun time” [(20), pp. 152–162]. For example, athletes in Australia are taken as using the time zone of the capital city, Canberra (UT +10 h). For the 1984 Olympic Games in Los Angeles, the time zone of the host city is (UT −8 h); the time zone difference is not 18 h, but the absolute value of −6 = 6 h.

#### Easterly direction

As discussed earlier, the direction of flight may have an impact on performance, with easterly travel likely to have more of an impact [(20), pp. 136, 138]. A dummy variable was used, where traveling east = 1, west or same time zone = 0.

#### Population (national)

Population is used as a factor in almost all of the predictive models for Olympic performance and is included here. Data were taken from Google Public Data (34).

#### GDP (national)

Nations with a high GDP are more likely to be able to direct resources to Olympic sports than nations with a low GDP. GDP data were taken from Google Public Data.

#### Distance (km)

We used great-circle distance using Excel to measure distance between the capital city of the country and the host nation (35).

#### Host nation

The potentially positive effect of being the host nation has been included as a factor, as it has with most other models predicting Olympic performance (15, 36–39). In the data, host nation = 1 and not host nation = 0.

#### Host previously

Whether the country was hosting the previous Olympics was coded as a dummy, previous host nation = 1 and not previous host nation = 0 (37, 38).

#### Host following

Hosting countries know many years before that they will host the Games and this may potentially affect preparation and support, so a measure is included regarding the host in the following 4 years, host nation next games = 1 and not host nation next games = 0 (37, 38).

## Results

The descriptive statistics and correlations for the variables are listed below in Table 2. The Pearson correlations show some interesting preliminary insights. The bivariate correlation between absolute time zone differences and medal performance is consistently negative, albeit often not significant for gold medal count (*r* = −0.029, *p* = 0.17), silver medal count (*r* = −0.012, *p* = 0.22), bronze medal count (*r* = −0.015, *p* = 0.21), and total medal count (*r* = −0.021, *p* = 0.19). Flying east seems to have a stronger negative effect for gold medal count (*r* =0.069, *p* = 0.08), a close to significant correlation with silver count (*r* = −0.044, *p* = 13), and bronze medal count (*r* = −0.066, *p* = 0.09), and total medal count (*r* = −0.064, *p* = 0.09). Being a host is generally very supportive for medal wins and anticipation or past hosting seem to be positive as well. This is particularly true for gold medals (*r* = 0.327, *p* < 0.001). As expected, population size and GDP are significantly and positively correlated with medal counts of all colors.

**Table 2:** Descriptive statistics and Pearson correlation matrix.

		**Mean**	**S.D**.	**1**	**2**	**3**	**4**	**5**	**6**	**7**	**8**	**9**	**10**	**11**	**12**
1.	Time zone difference	5.138	3.635	1											
2.	easterly direction	0.378	0.486	0.273***	1										
3.	Host?	0.053	0.225	−0.316***	−0.185**	1									
4.	Host previous?	0.053	0.225	0.141**	−0.087	−0.056	1								
5.	Host following?	0.053	0.225	0.128**	0.109*	−0.056	−0.056	1							
6.	Distance (km)	7,342.575	4,915.073	0.871***	0.250***	−0.329***	0.133**	0.092	1						
7.	Population (national)	120 million	260 million	0.066	−0.020	0.087	0.093	0.088	0.041	1					
8.	GDP (national)	1,491 billion	27,161 billion	0.060	0.019	0.052	0.123***	0.030	0.067	0.343***	1				
9.	Gold medals (total)	10.941	12.173	−0.029	−0.069	0.327***	0.183***	0.087	−0.021	0.456***	0.598***	1			
10.	Silver medals (total)	10.128	9.185	−0.012	−0.044	0.237***	0.173***	0.079	−0.010	0.383***	0.602***	0.894***	1		
11.	Bronze medals (total)	10.995	8.645	−0.015	−0.066	0.135**	0.146**	0.121**	0.005	0.342***	0.605***	0.811***	0.835***	1	
12.	Total medals	32.064	28.468	−0.021	−0.064	0.257***	0.178***	0.100*	−0.011	0.422***	0.634***	0.962***	0.958***	0.920***	1

* *p* < 0.05,

** *p* < 0.01,

*** *p* < 0.001.

The descriptive statistics show that the average number of time zones crossed in the sample is much higher (M = 5.14 time zones) than, for example studies, that study U.S. sporting teams traveling across the U.S. The odds of a team flying east are 37.8%. There is a 5.3% chance that country in the data sample hosts the Olympic Games. The average distance that a team travels is 7,342 km and given that China is in the sample, the average population is high (120 million). The average team in the sample won 10.9 gold medals, 10.1 silver medals, and 11.0 bronze medals (32 medals in total) *per* Olympic Games.

Table 3 shows the results from the multivariate moderated regression analysis for different team medal performance counts. All the key predictors of national team medal performance are included, including country dummies that can account for country heterogeneity (one country, the Netherlands, was taken as the baseline and omitted from the analysis as was the China dummy due to multicollinearity with population size). Where there is an interaction, the variables were first mean centered to reduce multicollinearity (61). Variance inflation factors are acceptable and around 1—and up to around 2 if distance in km—was included. In addition, we ran models with or without distance (km) and the results were similar.

**Table 3:** Multiple regression results for medal tallies.

	**Model 1**			**Model 2**			**Model 3**			**Model 4**		
	**Bronze medal tally**			**Bronze medal tally**			**Silver medal tally**			**Silver medal tally**		
	**β**	***t***	***p value***	**β**	***t***	***p value***	**β**	***t***	***p value***	**β**	***t***	***p value***
Constant		3.760	< 0.001***		2.332	0.011**		3.051	0.002***		1.902	0.030**
**Temporal factors**												
Time zone difference	−0.067	−0.794	0.215	−0.071	−0.837	0.202	−0.020	−0.250	0.402	−0.010	−0.122	0.452
easterly direction	−0.053	−1.308	0.097*	−0.045	−1.053	0.147	−0.021	−0.536	0.297	−0.044	−1.090	0.139
Time zone difference × easterly direction	–	–	–	−0.031	−0.722	0.236	–	–	–	0.083	2.047	0.021**
**Location factors**												
Host nation	0.087	2.068	0.020**	0.094	2.176	0.016**	0.174	4.309	< 0.001***	0.155	3.765	< 0.001***
Host previously?	0.093	2.353	0.010**	0.094	2.368	0.010**	0.100	2.656	0.005***	0.098	2.629	0.009***
Host following?	0.105	2.665	0.004***	0.107	2.700	0.004**	0.035	0.928	0.178	0.031	0.812	0.209
Distance (km)	0.049	0.555	0.290	0.048	0.535	0.297	0.027	0.321	0.375	0.032	0.379	0.353
**Economic factors**												
Population (national)	0.226	4.548	< 0.001***	0.225	4.507	< 0.001***	0.259	5.446	< 0.001***	0.263	5.579	< 0.001***
GDP (national)	0.236	4.386	< 0.001***	0.230	4.239	< 0.001***	0.145	2.809	0.003***	0.159	3.090	0.001***
**Country controls**												
AUS	0.150	2.661	0.005***.	0.149	2.645	0.005***	0.124	2.296	0.012**	0.125	2.352	0.010**
CAN	0.031	0.616	0.270	0.026	0.508	0.306	0.000	0.002	0.499	0.014	0.288	0.387
FRA	0.138	2.722	0.004***	0.138	2.725	0.004***	0.082	1.698	0.046**	0.081	1.694	0.046**
GER	0.297	6.578	< 0.001***	0.300	6.606	< 0.001***	0.202	4.674	< 0.001***	0.194	4.517	< 0.001***
GBR	0.143	2.786	0.003***	0.137	2.631	0.005***	0.116	2.365	0.010**	0.132	2.685	0.004***
HUN	0.103	2.051	0.021**	0.104	2.054	0.021**	0.089	1.833	0.035**	0.088	1.834	0.034**
ITA	0.101	1.995	0.024**	0.101	1.984	0.025**	0.054	1.105	0.136	0.055	1.137	0.129
JPN	0.062	1.211	0.114	0.061	1.180	0.120	0.002	0.046	0.482	0.006	0.127	0.450
RUS	0.422	9.857	< 0.001***	0.421	9.810	< 0.001***	0.331	8.083	< 0.001***	0.334	8.229	< 0.001***
KOR	0.017	0.331	0.371	0.014	0.282	0.390	0.019	0.400	0.345	0.026	0.539	0.295
ESP	−0.095	−1.869	0.032**	−0.096	−1.880	0.031**	−0.042	−0.849	0.199	−0.040	−0.816	0.208
UKR	0.104	2.390	0.009***	0.104	2.385	0.009***	0.018	0.436	0.332	0.018	0.446	0.328
USA	0.504	8.521	< 0.001***	0.502	8.460	< 0.001***	0.624	11.011	< 0.001***	0.630	11.207	< 0.001***
N	187			187			187			187		
*F* value	25.627			24.415			28.687			28.099		
Sign.	< 0.001***			< 0.001***			< 0.001***			< 0.001***		
*R* ^2^	0.764			0.765			0.784			0.789		

* *p* < 0.05,

** *p* < 0.01,

*** *p* < 0.001.

The results in Table 4 show a similar picture to the correlation table. Generally, there is a negative relationship between (1) absolute time zone difference and performance and (2) flying east and performance. There is a notable negative relationship between crossing more time zones and gold medal tally (*b* = −0.111, *p* = 0.06). This shows that there is an impact from time zones in the expected direction that is not mitigated by athlete preparation or other counter measures (more on this when future research is discussed). So, while some individual athletes will be less affected than others, there is a measurable and negative relationship between flying across more time zones and gold tally, even after controlling for distance. With respect to the flying direction, there is a notable relationship between traveling east and the bronze tally (*b* = −0.053, *p* = 0.10). Interestingly, the overall tally also suffers from flying in an easterly direction all else equal (*b* = −0.042, *p* = 0.10).

**Table 4:** Multiple regression results for medal tallies.

	**Model 5**			**Model 6**			**Model 7**			**Model 8**		
	**Gold medal tally**			**Gold medal tally**			**Total medal tally**			**Total medal tally**		
	**β**	***t***	***p value***	**β**	***t***	***p value***	**β**	***t***	***p value***	**β**	***t***	***p value***
Constant		1.901	0.030**		0.309	0.379		0.621	< 0.001***		1.834	0.034*
**Temporal factors**												
Time zone difference	−0.111	−1.577	0.059*	−0.110	−1.560	0.061*	−0.074	−1.155	0.125	−0.072	−1.112	0.134
easterly direction	−0.030	−0.884	0.189	−0.032	−0.890	0.188	−0.036	−1.151	0.126	−0.041	−1.272	0.103
Time zone difference × easterly direction	–	–	–	0.005	0.154	0.439	–	–	–	0.020	−0.604	0.274
**Location factors**												
Host nation	0.276	7.840	< 0.001***	0.275	7.573	< 0.001***	0.201	6.235	< 0.001***	0.196	5.918	< 0.001***
Host previously?	0.113	3.438	< 0.001***	0.113	3.423	< 0.001***	0.109	3.620	< 0.001***	0.109	3.597	< 0.001***
Host following?	0.046	1.408	0.081*	0.046	1.392	0.083*	0.063	2.095	0.019	0.062	2.051	0.021**
Distance (km)	0.157	2.118	0.018**	0.157	2.115	0.018**	0.091	1.341	0.091	0.092	1.355	0.089
**Economic factors**												
Population (national)			< 0.001***	0.313	7.523	< 0.001***	0.286	7.545	< 0.001***	0.287	7.549	< 0.001***
GDP (national)			0.018**	0.096	2.113	0.018**	0.159	3.875	< 0.001***	0.162	3.913	< 0.001***
**Country controls**												
AUS	0.013	0.269	0.395	0.013	0.270	0.393	0.091	2.115	0.018	0.091	2.120	0.018**
CAN	−0.059	−1.392	0.083*	−0.058	−1.353	0.089*	−0.016	−0.405	0.343	−0.012	−0.316	0.377
FRA	0.051	1.218	0.113	0.051	1.213	0.114	0.090	2.341	0.010	0.090	2.330	0.011**
GER	0.179	4.747	< 0.001***	0.179	4.700	< 0.000***	0.232	6.731	< 0.001***	0.230	6.637	< 0.001***
GBR	0.067	1.576	0.059*	0.069	1.575	0.059*	0.110	2.804	0.003	0.114	2.859	0.003**
HUN	0.090	2.129	0.018**	0.090	2.121	0.018**	0.098	2.554	0.006	0.098	2.545	0.006***
ITA	0.055	1.310	0.096*	0.055	1.307	0.097*	0.072	1.854	0.033	0.072	1.857	0.033**
JPN	0.012	0.273	0.393	0.012	0.279	0.391	0.025	0.628	0.266	0.026	0.651	0.258
RUS	0.278	7.781	< 0.001***	0.278	7.758	< 0.001***	0.354	10.836	< 0.001***	0.355	10.830	< 0.001***
KOR	0.013	0.318	0.376	0.014	0.327	0.372	0.017	0.442	0.330	0.019	0.481	0.316
ESP	−0.067	−1.575	0.059*	−0.067	−1.567	0.060*	−0.071	−1.824	0.035	−0.071	−1.808	0.036*
UKR	0.021	0.577	0.283	0.021	0.575	0.283	0.047	1.398	0.082	0.047	1.397	0.082*
USA	0.612	12.388	< 0.001***	0.612	12.343	< 0.001***	0.616	13.645	< 0.001***	0.617	13.632	< 0.001***
*N*	187			187			188			187		
*F* value	40.268			38.213			49.723			47.298		
Sign.	< 0.001***			< 0.001***			< 0.001***			< 0.001***		
*R* ^2^	0.836			0.836			0.863			0.863		

* *p* < 0.05,

** *p* < 0.01,

*** *p* < 0.001.

In line with the consistent negative signs across all time zone and direction coefficients, it is fair to say that (1) time zones affect performance negatively, especially in the gold tally (H1 is supported for gold tally and not rejected for the other medal types given the consistent negative pattern in the data), and (2) flying in easterly direction affects performance negatively, especially for bronze and total medals (H2 is supported for bronze and total (*p* <0.10) and not rejected for the other medal types because of the consistent negative pattern). In sum, evidence for H1 and H2 is mixed with only significant relationships for select medal metrics only. However, there is generally directional support across all medal metrics.

When the interaction between absolute time zone difference and flying east is included (61), it shows a significant negative coefficient for the interaction on silver medal count (*b* = 0.083, *p* = 0.021). This is not the case for gold, bronze and total medals. Generally, this result shows that there is a measurable weak negative relationship across the medals and that this pattern is visible after controlling for distance traveled, and country characteristics and hosting. Interestingly silver is behaving somewhat differently than gold and bronze.

**Figure 1:**
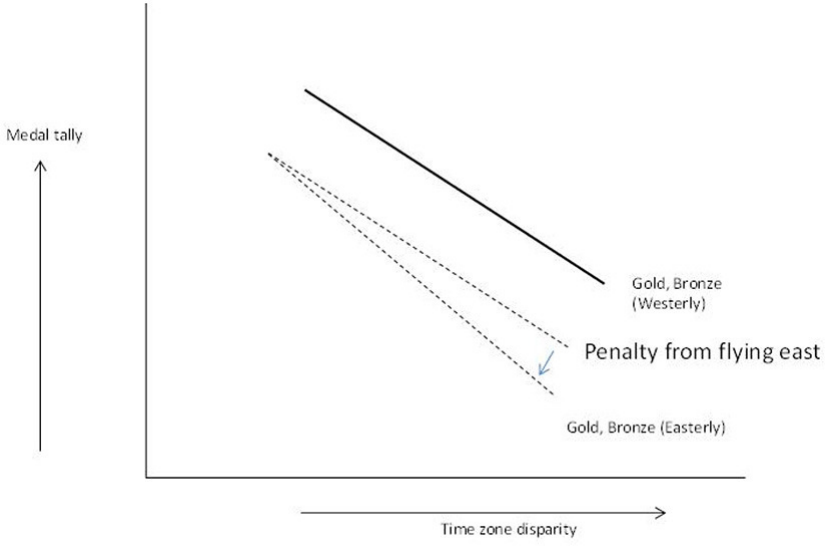
Gold and Bronze medal pattern.

**Figure 2:**
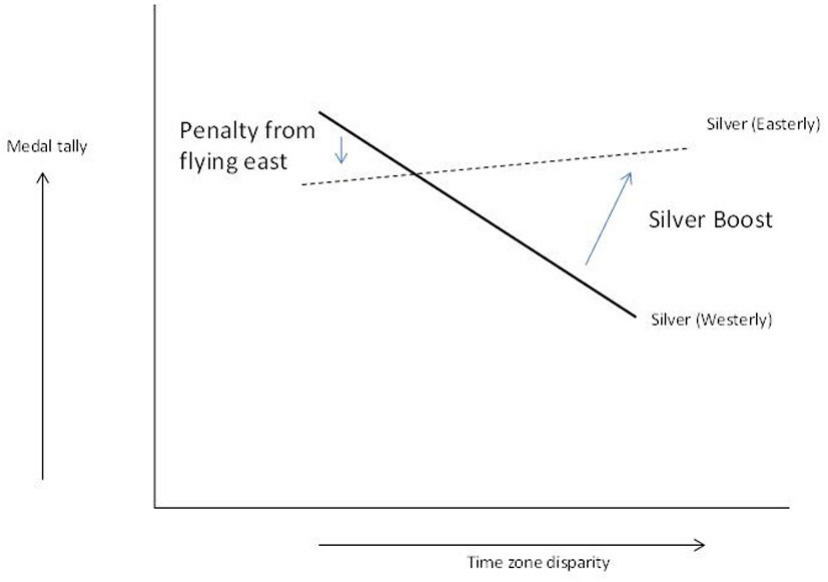
Silver medal pattern.

The fact that the interaction between time zones and direction is significant for silver medals warrants a deeper investigation in the nature of the interaction between crossing time zones and flight direction. First, Figure 1 shows the two *in*significant interactions (Gold and Bronze tally) indicating that large time zone differences are negative for performance and that the *slopes* are not statistically different for flying in an easterly or westerly direction (i.e., no significant acceleration downwards associated with direction although the slope is slightly more negative for the gold and bronze tally when flying in an easterly direction). There is generally a negative effect from flying in an easterly direction as discussed, and H2 is supported for gold and bronze. Second, the pattern is different for silver medals (Figure 2). For silver, the similar slopes are *not* similar, and a crossover emerges with for small time zone disparity a decrease in medal tally, but for high time zone disparity an increase in silver tally emerging. The increase in silver medal kicks in when the team both crosses multiple time zones *and* crosses them in an easterly direction. One explanation is that the gold medal count is demoted as the time zones crossed in easterly direction take their toll on gold medal performance more than on silver medal performance. If the silver medal count was affected equally, the bronze medal count would benefit in the same way, which is not the case, and the bronze pattern is basically negative as can be expected from crossing more time zones in easterly direction. This supports H3.

In sum, the slopes in Figures 1, 2 are generally negative, which is what can be expected from what is known about performance impairment from jet lag and easterly flight direction. However, the silver medal counts are impacted somewhat differently. At first glance, the silver tally seems to slightly benefit from crossing more time zones and doing so in easterly direction (=worst jet lag situation!). Although this may seem strange, this can be explained by peak performance impairment resulting in a boost to silver medal counts from decreased gold medal counts which are diminished more when athletes cross multiple time zones flying eastwards. Interestingly, the silver tally is lower in the low jet lag region of the graph (left hand side of Figure 2) if the flight direction is eastwards (the dotted line in Figure 2). In other words, performance impairment is not homogenous across the performance curve, and peak performers seem to struggle most from crossing multiple time zones, and the resultant jet lag, and flight direction. H3 is supported. To the best of our knowledge, this is the first study that theorizes on and finds empirical proof of the “gold demotion” effect that Olympic teams face, even with all the preparation and investments.

## Discussion

The basic assumption of this study is that organizations and employee performance management can learn from Olympic teams when it comes to travel, stress and health, and performance.

Olympic teams from well-resourced nations benefit from well-informed sports science and invest significant funds in the education and training of athletes in managing jet lag and other adverse health impacts from long distance travel. The management literature is calling for similar training and education programs for professional managers to enhance job performance and employee wellness (4). By studying the medal outcomes of Olympic teams, we can better understand the human experience and performance outcomes and then use this insight to inform future management practice (1).

The key findings of the study are that traveling across multiple time zones is indeed negative for performance and that flying east is particularly challenging for people. In the first instance, these findings are related to athletes because that is where the data comes from. Even for a highly performance managed setting as Olympic games, these effects are significant. As the number of time zones crossed increases, the negative effect on performance increases, in line with studies (9).

Flying eastwards increases the negative performance effect, as it is generally more difficult to adjust after a long-distance flight eastwards than it is to adjust to the equivalent long distance flight westwards (9, 24). This negative effect on performance is particularly visible in peak performance areas, namely gold medalists, where there is a “demotion effect” creating an unexpected boost to silver medals when athletes cross more time zones in easterly direction.

It is fair to presume that for the “amateurs” and unsupported team members in most organizations that have adopted global teams, these effects may be much larger. At the bare minimum, this study is highlighting a potential issue in management that is not receiving enough attention.

### Managerial implications

This study has significant implication for people traveling internationally and for scholars interested at public health in general. Athletes are well-informed about arriving early, the role of daylight, and therapeutic options to regulate the circadian rhythm but the stress and health effects seem material, even after some time. An interesting observation was a *positive* association between silver medal tallies and jet lag, indicating a “demotion effect,” where athletes who would have otherwise won a gold medal came second. Being on the top of your game when jet lagged seems to be a key challenge. In addition, this may signal that there may be a larger public health problem in society at large that we are not understanding sufficiently. This will become more important as society opens up after the pandemic.

The implications for public health and management are significant, especially for high-performing individuals that need to bring their “A-game.” Managers and humans in general need to consider the potential adverse effects of crossing multiple time zones, with preparations such as arriving to the venue early and auctioning a detailed plan for overcoming jet lag in organizations [e.g., (8)]. This requires focused education and training of the human resource to ensure peak performance and good health (1). Substantial amounts of business travel result in a significant amount of stress (62), and the resultant sleep deficit can cause significant cognitive and physical impairment (17) creating public health concerns.

### Limitations and future research

This study has some important limitations that offer opportunities for future research. Firstly, while we included data from 15 Olympic Games, future research can increase the sample size, include Winter Games and also include smaller nations. Secondly, future research is needed at the individual level with more details on the individual, his or her preparation, their engagement with jetlag management, and the timing during the day of the event. Thirdly, the role of chronotype could be studied in more detail and if additional data on preparation becomes available, more managerial insights can be obtained on what regimes help mitigate the impact on human sports performance. The time of day will have an impact as well for specific individuals. Overall, our study is still relevant as many of these factors should be averaged out by the large numbers of athletes participating in our study. Fourthly, while it is known that flying east has more negative effects than flying west on performance, the underlying mechanisms are poorly understood. While jet lag typically occurs after crossing three or more time zones (9), the more time zones crossed, the greater the extent of jet lag but the impact on the nature of the impairment and its duration is poorly understood and needs more research as well.

## Data availability statement

The original contributions presented in the study are included in the article/supplementary material, further inquiries can be directed to the corresponding author/s.

## Author contributions

All authors listed have made a substantial, direct, and intellectual contribution to the work and approved it for publication.

## Conflict of interest

The authors declare that the research was conducted in the absence of any commercial or financial relationships that could be construed as a potential conflict of interest.

## Publisher's note

All claims expressed in this article are solely those of the authors and do not necessarily represent those of their affiliated organizations, or those of the publisher, the editors and the reviewers. Any product that may be evaluated in this article, or claim that may be made by its manufacturer, is not guaranteed or endorsed by the publisher.
